# Toward a dimensional model of risk and protective factors influencing children's early cognitive, social, and emotional development during the COVID‐19 pandemic

**DOI:** 10.1111/infa.12495

**Published:** 2022-08-22

**Authors:** Alexandra Hendry, Shannon P. Gibson, Catherine Davies, Michelle McGillion, Nayeli Gonzalez‐Gomez

**Affiliations:** ^1^ Department of Experimental Psychology University of Oxford Oxford UK; ^2^ Department of Psychology Oxford Brookes University Oxford UK; ^3^ School of Languages, Cultures and Societies University of Leeds Leeds UK; ^4^ Department of Psychology University of Warwick Coventry UK

## Abstract

Variation in infants’ home environment is implicated in their cognitive and psycho‐social development. The pandemic has intensified variations in home environments through exacerbating socioeconomic inequalities, and increasing psychological stressors for some families. This study investigates the effects of parental (predominantly maternal) mental health, enriching activities and screen use on 280 24‐ to 52‐month‐olds’ executive functions, internalising and externalising problems, and pro‐social behaviour; with socioeconomic status and social support as contextual factors. Our results indicate that aspects of the home environment are differentially associated with children’s cognitive and psycho‐social development. Parents who experienced sustained mental distress during the pandemic tended to report higher child externalising and internalising problems, and executive function difficulties at follow‐up. Children who spent more time engaged in enriching activities with their parents showed stronger executive functions and social competence six months later. Screen use levels during the first year of the pandemic were not associated with outcomes. To mitigate the risk of persistent negative effects for this ‘pandemic generation’ of infants, our study highlights the importance of supporting parents’ mental health. As our results demonstrate the impact of social support on mental health, investing in support services and interventions promoting building support networks are likely to be beneficial.

## 1 INTRODUCTION

The developing human brain is experience‐driven; responding rapidly to enriching inputs and to psychological threats (DiPietro, 2000; Johnson et al., 2016; McLaughlin & Gabard‐Durnam, 2021). In the first 3 years of life, in particular, variation in the home environment has been linked to a range of outcomes, including social skills, cognitive function, and emotional well‐being (Fay‐Stammbach et al., 2014; Stack et al., 2010). In the UK, and many other countries, the COVID‐19 pandemic has created a context in which variation in infants' home environments has intensified: socioeconomic inequities have been exacerbated, and psychological stressors have been magnified within some families—for example due to the demands of working from home while providing childcare, financial and health worries, or the impacts of lack of access to support networks—while others remain well shielded (Di Gessa et al., 2021; Patel et al., 2020). Meanwhile, access to external provision of Early Childhood Education and Care—historically a buffer of external developmental risk factors—has been considerably constrained (Davies et al., 2021). These constraints on access to Early Childhood Education and Care, along with constraints on social interactions with nonimmediate family members, have also likely increased the extent to which parents are the primary influence on their child's development. Thus, as we seek to understand the impacts of the pandemic on young children, and identify for whom and how remediative support should be offered, we need to consider not only the multiple dimensions of family life, and how they are shaped by broader societal context, but also the multiple dimensions of psycho‐social and cognitive development, and the differential ways in which they are shaped by family life; this is the aim of the current study.

### 1.1 Dimensional models of early experience

One approach to examining the impacts of early experience on children's early cognitive, social, and emotional development is through dimensional models. Dimensional models seek to link variation in early experiences to mechanistic processes in order to characterize how different aspects of experience adaptively guide behavior (McLaughlin et al., 2020). A central principle of dimensional models is that different dimensions of experience will influence child development in at least partially distinct ways such that differential associations between inputs and outcomes can be used to generate insights into the different affective, cognitive, and neurobiological mechanistic processes that underlie those associations. In turn, this may inform intervention (McLaughlin et al., 2020). It is also worth noting, however, that diverse early experiences may influence childhood outcomes through shared mechanisms (McLaughlin & Sheridan, 2016), and that the same factor may be implicated in multiple pathways (see, e.g., the discussion of maternal mental health below).

Two pathways that have been hypothesized to influence child outcomes are an enrichment‐deprivation pathway, and a support‐threat pathway (also sometimes referred to as family investment and family stress, respectively) (McLaughlin et al., 2020; Vrantsidis et al., 2020). The enrichment‐deprivation pathway concerns the degree to which cognitively and socially stimulating inputs are received—or not—during development. The support‐threat pathway concerns the extent to which children are put under stress, and/or supported to cope with stress. Each pathway has many potential inputs, and the inputs that are particularly influential within a pathway will differ by population and context. Below we outline some potentially relevant inputs for infants and toddlers in the context of the COVID‐19 pandemic.

### 1.2 Parental mental health

The mental health of parents with young children appears to have been particularly affected by the pandemic (Racine et al., 2022); indeed, having young children was identified as a risk factor for distress during the pandemic (Pierce et al., 2020). Although the triggers of mental distress for parents during the pandemic are diverse, and vary from family to family, commonly reported factors include fears of infection, financial and job insecurity, the demands of balancing work and childcare, disruptions to self‐care routines, and low levels of social support (Alonzo et al., 2022; Calvano et al., 2021; Cheng et al., 2021; Davidson et al., 2021; Racine et al., 2022; Russell et al., 2020). It is well documented that parental mental health difficulties are a risk factor for early childhood difficulties with self‐regulation and related executive functions, as well as difficulties with coping with negative emotions or stressful situations, which may either manifest as a tendency to be self‐critical and anxious (i.e., internalizing difficulties) or a tendency to ‘act out’ in disruptive or aggressive ways (i.e., externalizing behavioral problems) (Carneiro et al., 2016; Power et al., 2021; Rigato et al., 2022; Ross et al., 2020; Stein et al., 2014; Sweeney & MacBeth, 2016). These effects may vary with demographic factors, with some evidence indicating that boys are especially vulnerable to the negative effects of parental mental distress in early and middle childhood, particularly in terms of externalizing problems (Choe et al., 2013; Cummings et al., 2005; Tronick & Reck, 2009; Wang & Yan, 2019).

Research has often considered the impacts of parental depression, anxiety, or stress in isolation, yet these frequently co‐occur such that associations attributed to one might include causes associated with the other (Stein et al., 2014). Chronicity (i.e., duration of exposure) of difficulties appears to play an important role in influencing child outcomes (Cornish et al., 2005; Hentges et al., 2020; Stein et al., 2014). In the context of the pandemic, where some initial acute distress in response to mass illness, loss of life, and social upheaval, followed by a process of adjustment and resilience might be considered an adaptive response, chronicity of mental distress may be particularly key in influencing child outcomes. However, the negative effects of parental mental distress do not seem to be limited to only extreme cases (Tronick & Reck, 2009), such that it is necessary to consider chronicity of mild mental distress alongside severe distress when evaluating impacts on child outcomes.

A key way in which parental mental health appears to impact early child development is via the support‐threat pathway. This may take two forms: Firstly, poor parental mental health may reduce the support that a parent is able to provide for their child's psycho‐social and cognitive development, by negatively impacting the quality of emotional inputs provided. Research indicates that parental mental distress lessens sensitivity (i.e., appropriate responding to a child's cues and emotional state) (Bernard et al., 2018; Ku & Feng, 2021). In turn, lower parental sensitivity within the first years of life is associated with higher child internalizing and externalizing problems (Cooke et al., 2022) and lower child executive functions (Blair, Raver, Berry, et al., 2014; Ku & Feng, 2021; Valcan et al., 2018). Secondly, poor parental mental health may increase the threat that the parent poses to their child. Poor parental mental health has been linked to increase in harsh parenting (i.e., verbal or physical aggression) before (Le et al., 2017; Masarik & Conger, 2017) and during the pandemic (Chung et al., 2020; Connell & Strambler, 2021). Harsh parenting has been positively associated with children's internalizing and externalizing problems (Berthelon et al., 2020; Gulenc et al., 2018) and negatively associated with children's executive functions (Valcan et al., 2018).

The second pathway by which poor parental mental health may lead to negative child outcomes is through enrichment‐deprivation. Specifically, poor parental mental health may lead to a reduction in the quality or quantity of cognitively stimulating experiences (Kiernan & Huerta, 2008; Lovejoy et al., 2000; Paulson et al., 2006). This pathway is discussed in more detail below.

### 1.3 Enriching activities

As previously described, the enrichment‐deprivation pathway concerns the degree to which cognitively and socially stimulating inputs are received—or not—during development. The activities that parents engage in with their child appear to be a particularly important input in early development. For example, parental provision of resources and activities (e.g., provision of books, encouragement of learning, and going on excursions) has been concurrently associated with child executive function at ages 5–6 years, and with growth in executive functions over time (Rosen et al., 2020), while parental responsiveness and cognitive stimulation predict change in executive function between the ages of 3–5 years (Blair, Raver, Berry, et al., 2014). In the specific context of the pandemic, more exposure to parent‐child enriching activities (e.g., talking, singing cooking, exercise…) during two periods of social distancing spanning a 6‐month period, is associated with higher cognitive executive functions, but not emotion regulation, among 15‐ to 36‐month‐olds (Hendry et al., 2022), and the greater the number of activities (excluding screen use, lessons, and physical play) engaged in during the pandemic, the higher 3‐ to 5‐year‐olds’ concurrent performance on an executive function task (Stucke et al., 2022). It is not yet known whether these effects will be sustained over a longer period, or whether the benefits of parent‐child enriching activities are specific to older toddlers compared with infants. To date, sex differences in these effects have not been reported (Blair, Raver, & Berry, 2014; Hendry et al., 2022; Rosen et al., 2020).

Engagement in parent‐child activities may also be expected to support the development of social competence (i.e., engagement in prosocial behaviors such as sharing readily and being considerate of others' feelings). Although this link is less well‐researched in early childhood, cognitive stimulation has been linked with social competence among 3‐ to 5‐year‐olds, across both boys and girls (McGroder, 2000). As the babies and toddlers of the pandemic enter group preschool settings, understanding the link between previous exposure to enriching parent‐child activities and their developing social competence may help Early Years practitioners and parents to implement supportive strategies.

### 1.4 Screen use

Evidence suggests that the pandemic triggered an increase in infant and toddlers' screen time across many countries globally (Bergmann et al., 2021), making this another important input to consider regarding associations between the home environment and developmental outcomes. Screen use has the potential to exert both positive and negative influence on cognitive development via an enrichment‐deprivation pathway. Age‐appropriate educational media may have a positive effect on children by enriching the home learning environment, providing opportunities for joint parent‐child engagement, discussion of moral themes and emotion recognition, and fine motor skill development (Barr, 2019; Mares & Pan, 2013; Padilla‐Walker et al., 2020; Souto et al., 2020; Vandewater & Bickham, 2004). Yet, excessive screen time is also widely considered to negatively impact several mechanisms, which have previously been implicated in executive function development: Screen time may reduce the quality and quantity of parent‐child interactions (Masur et al., 2016), disrupt the quantity or quality of infant sleep (Ribner et al., 2019; Staples et al., 2021), and reduce the opportunities for young children to learn to self‐regulate (Coyne et al., 2021). Indeed, longitudinal studies have found greater exposure to media and television in infancy and toddlerhood is predictive of lower executive function later in childhood (Cliff et al., 2018; McHarg et al., 2020). Cross‐sectional research also points to an important but less well‐studied link between screen use and social competence (Wan et al., 2021). During the specific context of the pandemic, the amount of time that parents reported that their child spent engaged with a screen showed a small negative association with child executive functions (Hendry et al., 2022); what is not yet known is whether these effects are pervasive over a longer period, particularly after taking into account other aspects of the home environment.

### 1.5 Contextual factors

At least some of the sources of variation in the environmental inputs described above may be attributable to socioeconomic inequality. In the UK, adults with lower incomes were more likely to experience mental health difficulties, both before and during the pandemic (Pierce et al., 2020) and maternal depression has been identified as a potential mediator of the association between socioeconomic status (SES) and child externalizing problems (but not internalizing problems) (Rutherford et al., 2019). Concerning variation in parent‐child enriching activities, although there is a broad literature linking socioeconomic factors to enrichment in the home (Hackman et al., 2015; Rosen et al., 2020; Vrantsidis et al., 2020), much of this work is based on the Home Observation of the Environment (HOME) measure, which is biased toward costly sources of enrichment (e.g., excursions, musical equipment provision), and overlooks other, free sources of enrichment such as singing and telling stories (Zaslow et al., 2006). When alternative measures were used, a pervasive association between SES and parent‐child enriching activities in the context of the pandemic was not found (Hendry et al., 2022), while US‐based research has demonstrated that many families provide a cognitively stimulating home environment despite economic hardship (DeJoseph et al., 2021). Meanwhile, infant and toddler screen use has been observed to be negatively associated with SES, with parents from higher‐SES reporting their children to have less screen time both before (Hendry et al., 2022; Ribner & McHarg, 2021; Supanitayanon et al., 2020) and during the pandemic (Bergmann et al., 2021; Hendry et al., 2022) compared with parents from less‐advantaged backgrounds.

Conversely, access to social support may buffer the negative effects of the pandemic on the home environment. Multiple studies have found perceived social support to be negatively associated with maternal mental health difficulties (Camisasca et al., 2021; Chang, 2017; Hetherington et al., 2018). Moreover, during the pandemic, social support has been linked to lower levels of depression in US mothers (Gustafsson et al., 2021) and to moderate the relation between repetitive negative thinking and depression in UK mothers of infants during the pandemic (Harrison et al., 2021). It has also been demonstrated, in a nonpandemic context, that social support positively impacts early child cognitive development via an enrichment‐deprivation pathway, in that greater provision of cognitively enriching activities and resources mediates an association between social support and child language skills (Chang, 2017). During the pandemic, social support was associated with higher levels of positive experiences in parent‐child relationships among Polish parents (Gambin et al., 2020).

### 1.6 The current study

**FIGURE 1:**
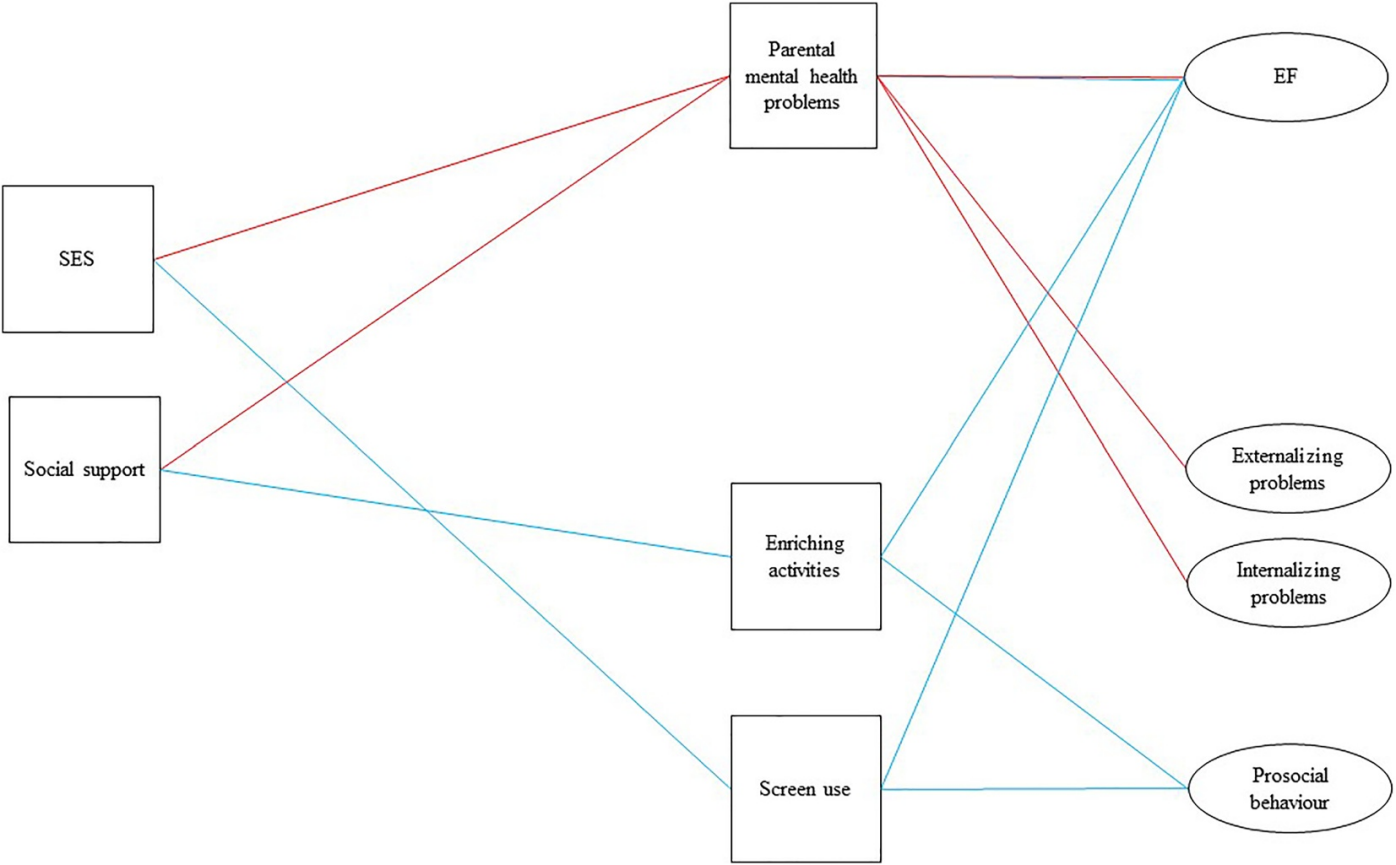
Conceptual model illustrating the hypothesized effect of SES and social support on child outcomes, via parental mental health problems, enriching activities, and screen use. Blue lines are considered to indicate an enrichment‐deprivation pathway, and red lines, a support‐threat pathway

Our aim was to investigate how aspects of children's domestic experience are shaping their early development during the pandemic. By considering multiple dimensions of experience in terms of inputs and outcomes, we aim to provide an integrative account of development. Using data collected from 280 UK‐based families within the conceptual model outlined in Figure 1, we investigate the effects of parental mental health, enriching activities and screen use on child executive functions, internalizing and externalizing problems, and prosocial behavior. We hypothesize that these associations are implicated in two key pathways: a support‐threat pathway, and an enrichment‐deprivation pathway.

Data were collected in

- Spring 2020, during the first and most restrictive social distancing measures in the UK (when between 23rd March and 1st June nurseries and schools were closed to all but the children of front‐line key workers, and the most vulnerable children, and the public were prohibited from socializing with others outside of their immediate household)
- Winter 2020 during the second lockdown period between 5th November and 2nd December (when people were prohibited from meeting those not in their “support bubble” inside but were able to meet one person from outside their support bubble outdoors), and
- Spring 2021 roughly a year after the introduction of social distancing measures, when restrictions over indoor mixing of multiple households were still in place.

## 2 METHODS

### 2.1 Participants

Families with infants and children between 8 and 36 months of age from across the UK were recruited through online advertisements on research‐related websites and via social media to take part in the Social Distancing and Development project. The data reported in this study was collected in Spring 2020, Winter 2020, and Spring 2021 as shown in Table 1.

**TABLE 1:** Data collection points and sample size

Timepoint	Date	Valid *n*	Relevant questions for this study
Spring 2020	3 Mar–28 Jun 2020	502	SES Parental mental health Activities in the home
Winter 2020	27 Nov–18 Dec 2020	227	Parental mental health Activities in the home Perceived social support
Spring 2021	27 Apr–2 Jun 2021	280^a^	Parental mental health Perceived social support Child's cognitive and psycho‐social strengths and difficulties

^a^ Excluding participants younger than 24 months at the Spring 2021 data collection point (*n* = 51).

Only UK‐based infants with a gestational age of 37 weeks or over, no known genetic conditions, and aged over 24 months at the Spring 2021 data completion point (so that outcome measures would be suitable) are included in this study: see Supporting Information S1 (https://osf.io/69myq/) and discussion below for details of demographic differences between participants contributing data at each timepoint. Ninety‐eight per cent of respondents were the target child's mother, 2% their father.

Data from Spring 2020 and Winter 2020 relating to participant demographics, access to Early Childhood Education and Care, enriching activities, screen use, and executive functions have been previously reported in Hendry et al. (2022). No data relating to parental mental health, or social support at any timepoint has previously been reported, and no outcome data from the Spring 2021 data collection wave (i.e., executive functions, internalizing and externalizing problems, and prosocial behavior) has been previously reported. All procedures performed in this manuscript were in accordance with the 1964 Helsinki Declaration and its later amendments or comparable ethical standards. All participating caregivers provided informed consent at each timepoint, on behalf of themselves and their child. This study received ethics approval from the Oxford Brookes University Research Ethics Committee (UREC): ref 20023. On completion of the questionnaires, families were given £30 (Spring 2020), £5 (Winter 2020), and £10 (Spring 2021) Amazon vouchers.

### 2.2 Measures

#### 2.2.1 Parental mental health

At each timepoint, respondents were asked to complete the Depression Anxiety and Stress Scales (DASS‐21) (Lovibond & Lovibond, 1995). Respondents rated how much each of 21 statements relating to indicators of depression, anxiety, and stress applied to them over the past week, on a scale from 0 (*Not at all*) to 3 (*Most of the time*). Scores were multiplied by 2 for comparability with normative data derived from the 42‐item DASS. Internal consistency for the Depression, Anxiety and Stress scales at each timepoint ranged from Cronbach's alpha = 0.69–0.90. Following Hentges et al. (2020), for each timepoint, we identified whether or not the respondent scored in the range of clinical concern (mild to extremely severe) for depression, anxiety, or stress, to compute a dichotomous indicator of mental health difficulties. We then calculated the chronicity of mental health difficulties by totaling the dichotomized scores, for participants who contributed data at 3 timepoints (*n* = 195). For participants with only 2 timepoints (*n* = 81), if the dichotomized score was consistent at both timepoints we interpolated a third timepoint score to be identical to the other 2 timepoints (*n* = 56), and if the dichotomized score was inconsistent between the 2 timepoints we interpolated a third timepoint score as 0.5 (*n* = 25). Participants with DASS data for only 1 timepoint were treated as missing (*n* = 4). Forty‐five percent of respondents did not score in the range of clinical concern at any timepoint, 35% showed mental health difficulties at 1 or 2 timepoints, and 20% showed sustained mental health difficulties across all 3 timepoints.

#### 2.2.2 Enriching activities and screen use

In line with Kartushina et al. (2021) and Bergmann et al. (2021), and as previously described in Hendry et al. (2022), in Spring 2020 and Winter 2020, respondents were asked to report on the kinds of activities that they did with their child—for example, reading, singing, arts and crafts, and the frequency with which they did those activities. This was used to compute an Enriching Activities score as detailed in SM 1.2. In Spring 2020 and Winter 2020, respondents were asked to report on the frequency and duration that their child watched TV or played on a touchscreen. This was used to compute a Screen Use score as detailed in SM 1.2. We elected to use aggregate scores from Spring and Winter 2020 as these were the two primary lockdown periods in the UK, such that this period was a time in which the home environment can be expected to be especially influential to child development.

There was no significant difference in scores between participants who were retained through to Spring 2021 and those who were not retained with regard to Enriching activities (*t*(500) = −1.817, *p* = 0.070) or Screen use (*t*(500) = −1.644, *p* = 0.101).

#### 2.2.3 SES

At study entry, respondents provided data on four indices of SES:

1. Neighborhood deprivation index: Postcode data was used to compute an Index of Multiple Deprivation (IMD) decile group where 1 = most deprived, 10 = least deprived, using either the English (Noble et al., 2019), the Northern Ireland (Power & Green, 2019), the Welsh (Welsh Government, 2019) or the Scottish IMD (Scottish Government, 2020) as appropriate.
2. Income: Parents were asked to report their total household income from one of seven categories (see Table 2).
3. Parental education: Parents were asked to report their highest level of education completed. For participants with two parents, a mean parental education score was computed.
4. Parents' occupational prestige: Parents were asked to report the occupation for each parent. This was then converted into scores ranging from 1 (lowest status) to 9 (highest status) based on Hollingshead (1975). For participants with two working parents, a mean parental occupation score was computed.

Multiple imputation with 100 iterations was used to impute missing data (IMD *N* = 3, parental education *N* = 1, parental occupation *N* = 6). As described in Hendry et al. (2022) and SM 1.1 (https://osf.io/69myq/), Principal Components Analysis (PCA) was used to extract a single SES factor score from these four indices. As reported in Table 2, participants were socio‐economically diverse but skewed toward higher SES. There was no significant difference in overall SES between the sample retained to Spring 2021, and the sample originally recruited (*t*(859) = −1.016, *p* = 0.310).

**TABLE 2:** Descriptive data for participants

	n	Mean	SD	Median	Min	Max
Age in months Spring 2021	280	34.08	6.70	32.84	24.10	51.91
Home environment indicators						
Parental mental health (number of timepoints scoring in the clinical range on DASS)	276	1.13	1.18	1	0	3
2020 Pandemic Enriching Activities	280	0.04	0.93	0.07	−2.27	3.02
2020 Pandemic Screen Use	280	0.04	0.96	−0.04	−1.57	3.95
Contextual factors						
Socioeconomic Status (SES)	280	0.05	1.00	0.21	−2.58	2.01
Neighborhood deprivation^a^	280	6.71	2.64	7.00	1	10
Household Income^b^	280	4.85	1.91	5.00	1	7
Parental Education^c^	280	5.38	1.29	5.50	2	8
Parental Occupation^d^	280	6.98	1.56	7.50	3	9
2020–2021 Social support (MOS SSS)	280	78.38	19.70	82.89	0	100
Outcome measures						
EF strengths (EEFQ CEF)	176	5.14	0.71	5.20	2.62	6.62
EF difficulties (BRIEF‐P EMI)	280	40.32	8.78	39	27	71
Externalizing problems (SDQ)	280	5.66	3.30	5	0	18
Internalizing problems (SDQ)	280	3.33	2.87	3	0	13
Prosocial behavior (SDQ)	280	6.90	1.98	7	1	10

Abbreviations: BRIEF‐P, Behavior Rating Inventory of Executive Function—Preschool Version; CEF, cognitive executive function; DASS, Depression Anxiety and Stress Scales; EEFQ, Early Executive Functions Questionnaire; EF, executive function; EMI, Emergent Metacognition Index; MOS SSS, Medical Outcomes Study social support survey; SDQ, Strengths and Difficulties Questionnaire.

^a^ Index of Multiple Deprivation decile, where 1 = most deprived, 10 = least deprived.

^b^ Household income brackets: 1 = £0–£20k, 2 = £21k–£30k, 3 = £31k–£40k, 4 = £41k–£50k, 5 = £51k–£60k, 6 = £61k–£70k, 7 = £71k or over.

^c^ Categories of highest level of education completed: 1 = Primary school, 2 = Secondary school, 3 = Sixth form or college, 4 = Vocational college, 5 = Undergraduate, 6 = Postgraduate, 7 = MBA, 8 = Doctoral degree.

^d^ Occupational prestige where 1 = lowest prestige, 9 = highest prestige.

#### 2.2.4 Social support

In Winter 2020 and Spring 2021, participants were presented with the Medical Outcomes Study social support survey (Sherbourne & Stewart, 1991), which asks respondents how often during the last 6 months each of 19 examples of types of social support—relating to sources of advice, practical support, emotional connection, and friendship—was available to them, if needed, on a scale from None of the time (1) to All of the time (5). Internal consistency of ratings of support across the different types of support was high (Cronbach's alpha = 0.97 in Winter 2020 and Spring 2021). Following Sherbourne and Stewart (1991), at each timepoint for each participant, we computed the average of all the item scores, subtracted the minimum possible score and divided this value by the maximum possible range. The resultant value was multiplied by 100 to yield a Social support score, which was then standardized within timepoint. Standardized social support scores showed high stability across timepoints (*r* = 0.703, *p* < 0.001), and were therefore averaged to compute a 2020–2021 Social support score (if only T4 data was available (*n* = 87) this score was used instead). Given the reporting span of the questionnaire (i.e., the last 6 months) the 2020–2021 Social support score can be considered to apply to the entire Spring 2020 to Spring 2021 period.

#### 2.2.5 Executive functions

In Spring 2021, respondents were asked to complete the Early Executive Functions Questionnaire (EEFQ) (Hendry & Holmboe, 2021) (if the target child was 36 months or under) and/or the Behavior Rating Inventory of Executive Function‐Preschool Version (BRIEF‐P) (Gioia et al., 2002) (if the target child was 24 months or over).

##### EEFQ

The EEFQ comprises 31 items relating to the control of attention, behavior, and emotion (Hendry & Holmboe, 2021). A Cognitive Executive Functions (CEF) factor with loadings from 24 items relating to inhibitory control, working memory, and cognitive flexibility has previously been demonstrated to show partial measurement invariance by age for 8‐ to 36‐month‐olds, in a partially overlapping sample to this study (Hendry et al., 2022). The EEFQ also comprises seven items relating to the control of emotion (‘Regulation scale’), which were not included here due to high conceptual overlap with the SDQ scales.

Parents are asked to report on a 7‐item scale how often their child has exhibited a particular behavior during the preceding fortnight—or, for behaviors that may be uncommon in all children, or highly context‐dependent, play a short game with their child designed to elicit a particular skill and then report back on their child's performance (three items). To compute a composite CEF score, the mean of items from the Inhibitory Control, Working Memory, and Flexibility subscales can be calculated, where a minimum of 70% of eligible items are complete, to give a score ranging from 1 to 7. Internal consistency was excellent for the CEF composite (Cronbach's alpha = 0.87) but was reduced by low item‐total correlations for two items (WM6 and WM game); these items were removed before computing the CEF composite. For the path analysis, items were loaded individually onto a CEF latent factor (see Analytic strategy).

##### BRIEF‐P

The BRIEF‐P measures executive functioning through observer report in children aged 24 months and above. Respondents were also asked to complete this measure in Winter 2020 if their child had already reached 24 months of age but as only 44% of the current sample met this criterion, only the Spring 2021 data are reported here. The BRIEF‐P is a 63‐item questionnaire in which respondents are asked to report on the extent to which a given statement for example, “Needs help from an adult to stay on task”, is true for the target child on a 3‐item scale (1 = Never, 2 = Sometimes, and 3 = Often). High scores correspond to greater difficulties. Items are grouped into five scales, which are combined to form 3 broader indexes: Inhibitory Self‐Control (Inhibit and Emotional Control scales), Flexibility (Shift and Emotional Control scales), and Emergent Metacognition (Working Memory and Plan/Organize scales). Items in the Emergent Metacognition index specifically capture the more cognitive aspects of executive function (e.g., Has trouble concentrating on games, puzzles, or play activities) and thus was selected for equivalence with the EEFQ‐CEF, and to complement the psycho‐social behaviors captured by the SDQ. Due to the high prevalence of executive function difficulties among children with ADHD and or oppositional‐defiant disorder traits (who would be expected to have high SDQ Externalizing problems scores), we expect moderate‐to‐high associations between BRIEF‐P Emergent Metacognition and SDQ Externalizing Difficulties scores (Ezpeleta & Granero, 2015). Items for the Emergent Metacognition index are summed such that the range is 27–81. Composite scores were positively skewed, so were log‐transformed prior to analysis. For the path analysis, items were loaded individually onto a latent EMI factor (see Analytic strategy).

#### 2.2.6 Internalizing and externalizing problems, and prosocial behavior

In Spring 2021, parents of 2‐ to 4‐year‐olds were asked to report on their child's psycho‐social well‐being using the Strengths and Difficulties Questionnaire (SDQ) (Goodman, 1997). The SDQ is a 25‐item behavioral screening questionnaire that asks the extent to which a given statement for example, “Nervous or clingy in new situations”, is true for the target child on a 3‐item scale (0 = Not true, 1 = Somewhat true, and 2 = Certainly true). The items are divided between 5 scales but in low‐risk or general population samples such as ours, authors advise using a three‐subscale division of the SDQ: externalizing problems (conduct problems and hyperactivity/inattention scales); internalizing problems (emotional symptoms and peer relationship problems scales); and a separate indicator of social competence (prosocial scale) (Goodman et al., 2010). Internal consistency of these subscales ranged from Cronbach's alpha = 0.701 (Prosocial behavior) to 0.778 (externalizing problems). To compute a composite score, items for the subscales are summed (i.e., the range for the internalizing and externalizing scales = 0–20; for prosocial scale = 0–10). SDQ internalizing and externalizing composite scores were positively skewed, so were log‐transformed prior to analysis. For the path analysis, items were loaded individually onto separate latent factors for externalizing problems, internalizing problems, and prosocial behavior (see Analytic strategy).

### 2.3 Analytic strategy

Path analysis was conducted to test the conceptual model outlined in Figure 1, which was informed by the prior research summarized in the Introduction. Outcome variables were computed as latent factors using Structural Equation Modeling (SEM) to account for measurement error (see SM 1.4 for model comparisons). As one of our executive function measures was only suitable for children up to 36 months of age, we ran two separate models: Model 1, using EEFQ‐CEF items was run with 24‐ to 36‐month‐old participants; and Model 2, using BRIEF‐P EMI items was run with the full sample. To check that differences between the models were not attributable to sample size and age differences, in SM 1.5 we also report the results of Model 2 when only 2‐to 3‐year‐olds were included; key differences are highlighted in the results summary. To ascertain whether young children of a particular age or sex are particularly affected by variation in the home environment, for each significant regression pathway identified above between an aspect of the home environment and a child outcome, we ran exploratory tests for moderation effects of child age or sex.

SEM were conducted using the *lavaan* package vn 0.6‐7 in R (Rosseel, 2012). Missing data were dealt with using full information maximum likelihood estimation (FIML) with an expectation maximization algorithm. Model fit was assessed using the approximation (RMSEA), and standardized root mean square residual (SRMR). Values indicating good fit were less than 0.06 for the RMSEA, and less than 0.08 for the SRMR (Hu & Bentler, 1999). Values indicating adequate model were a RMSEA of 0.06–0.08, and SRMR less than 0.10 (Hu & Bentler, 1999; Kline, 2015). We report standardized estimates computed using *std.all.*

Descriptive and post‐hoc moderation analyses were conducted in *RStudio* (vn 1.2.5033) using composite scores as described above.

## 3 RESULTS

### 3.1 Descriptive data

**FIGURE 2:**
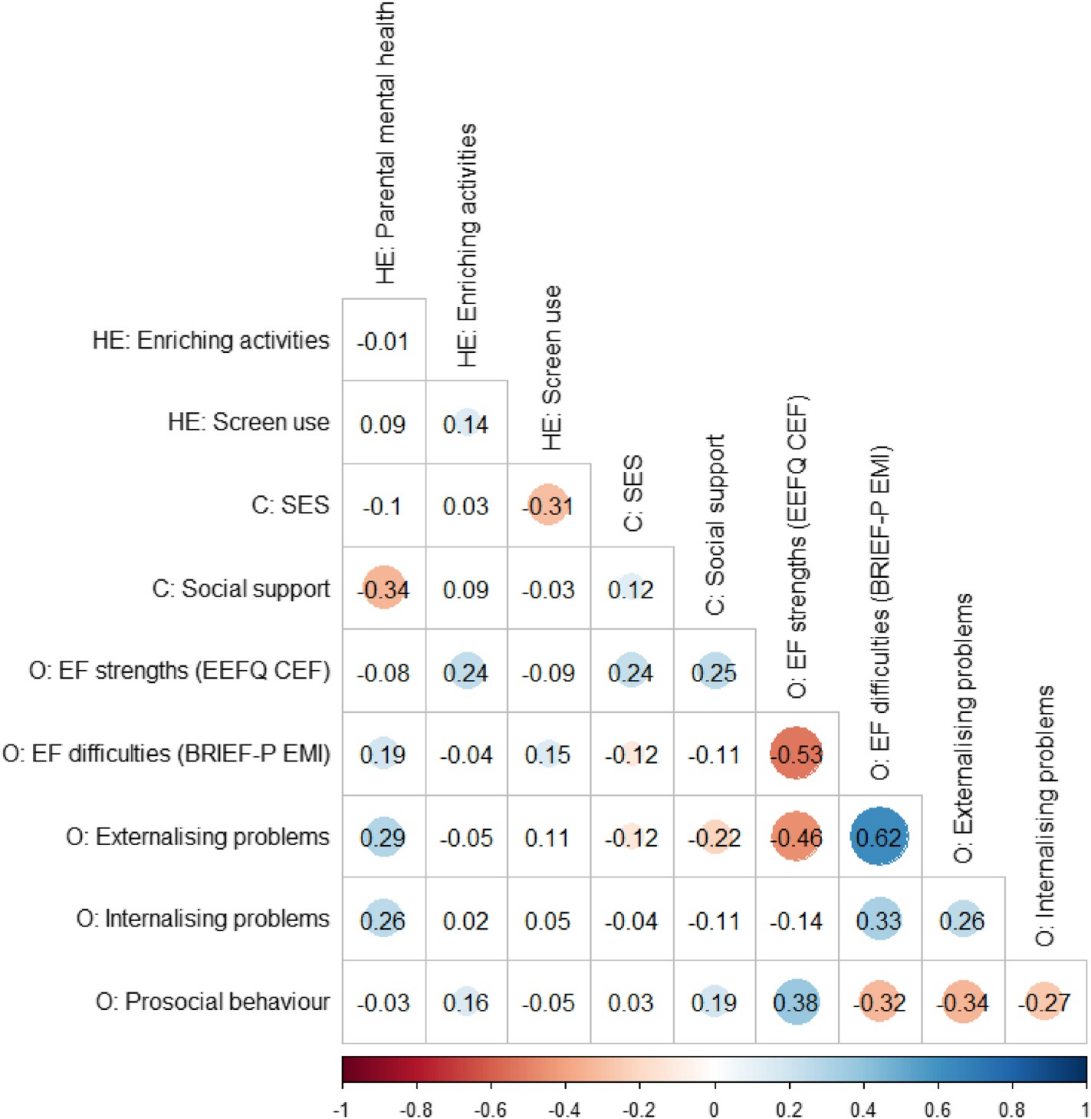
Pearson correlations between key study variables. BRIEF‐P, Behavior Rating Inventory of Executive Function – Preschool Version; C, Contextual variables; CEF, cognitive executive function; EEFQ, Early Executive Functions Questionnaire; EMI, Emergent Metacognition Index; HE, Home Environment variables; O, Outcome variables; *n* = 280 excluding correlations involving parental mental health (*n* = 276) or CEF (*n* = 174). Correlations significant at *p* < 0.05 are shown with a colored circle with size corresponding to the strength of association, and color corresponding to the direction of association (red = negative association, blue = positive association)

Summary descriptive data are presented in Table 2. SDQ and BRIEF‐P scores are contextualized with regard to comparison samples in SM 1.3. Correlations among these variables are presented in Figure 2. As shown in Figure 2, the association between SES and social support was not significant, indicating that these are distinct contextual factors. There was a weak negative association between enriching activities and screen use, but no significant association between parental mental health and enriching activities or screen use, indicating that these elements of the home environment are distinct. In terms of child outcomes, executive function (EF) strengths and EF difficulties showed a moderate negative association. EF difficulties showed a strong positive association with externalizing problems and a moderate positive association with internalizing problems. Prosocial behavior showed a moderate positive association with EF strengths, and weak‐to‐moderate negative associations with EF difficulties and internalizing and externalizing problems.

### 3.2 Path analysis

For clarity, in the description of results below, we discuss both models in terms of EF ability, which means that for Model 2 associations from EF difficulties have been reversed.

A structural model with latent factors for each outcome indicated adequate model fit (see SM 1.4 for details) and so was taken forward to path analysis to test the conceptual model in Figure 1. Exploratory direct effects of SES and social support on outcomes were included in the model, and age and sex were included as covariates of each outcome. Model fit to the data was adequate to good: Model 1 RMSEA = 0.068, SRMR = 0.088; Model 2 RMSEA = 0.056, SRMR = 0.066.

**FIGURE 3:**
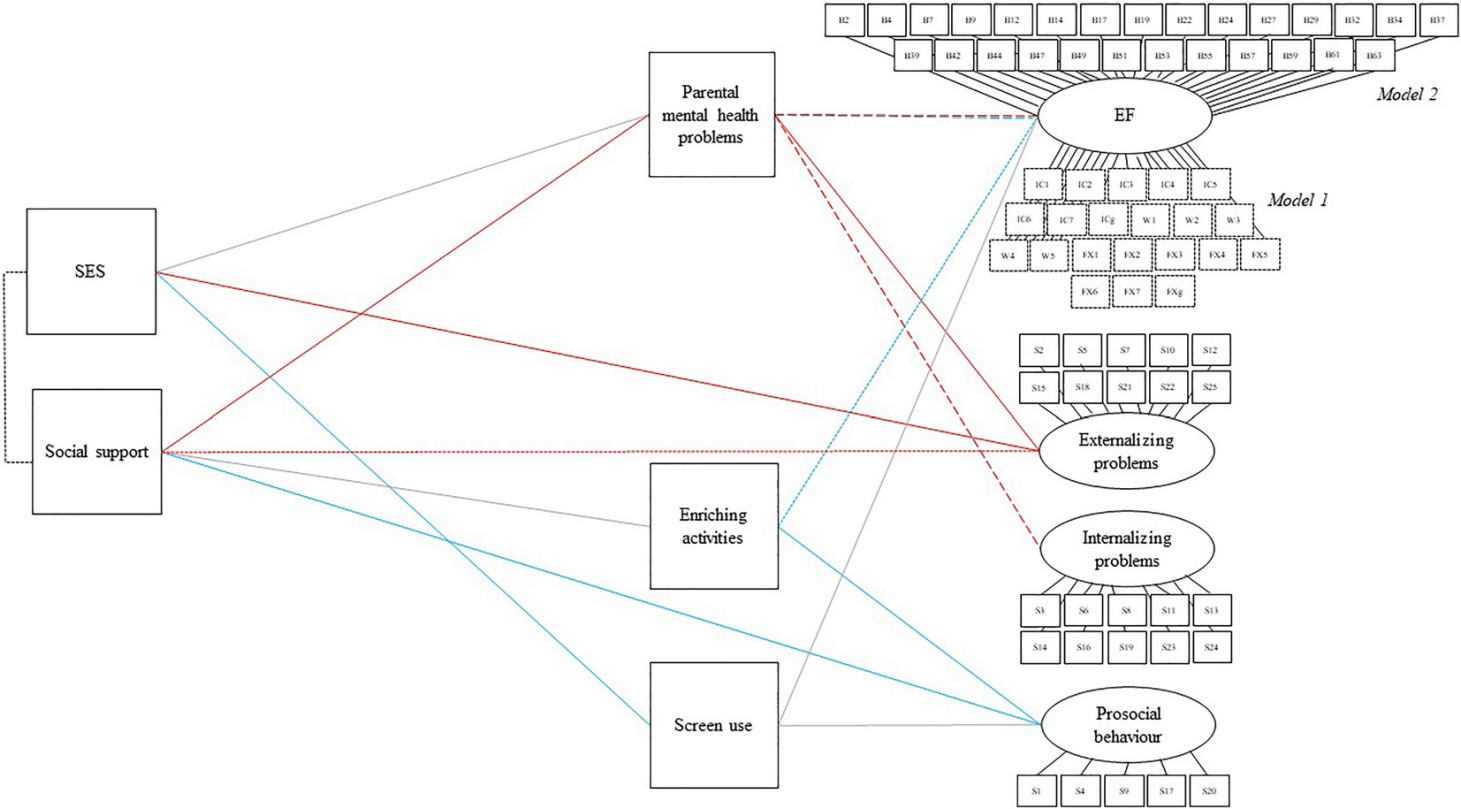
Path diagram illustrating the effect of SES and social support on child outcomes, via parental mental health problems, enriching activities, and screen use. Regression pathways significant at *p* < 0.05 in both Model 1 (EEFQ CEF data included as indicators of EF, sub‐sample of children 3 years and under, *n* = 177) and Model 2b (BRIEF‐P EMI data included as indicators of EF, full sample, *n* = 280) are shown with a solid‐colored line. Pathways significant for Model 1 only shown with a short‐dashed line, pathways significant for Model 2 only shown with a long‐dashed line. Blue lines are considered to indicate a cognitive‐enrichment pathway, and red lines a support‐threat pathway. Nonsignificant pathways are shown with a solid gray line. Error variances, age, and sex covariates are not shown

Model 1 explained 14% of the variance in child EF, and Model 2, 8%. As shown in Table 3 and Figure 3, for Model 2 but not Model 1 there was a small negative effect of parental mental health difficulties on EF. For Model 1 but not Model 2 there was a small positive effect of enriching activities on EF. Screen use did not show a significant association with EF in either model. There was no significant direct effect of SES on EF, and neither of the hypothesized indirect paths from SES to EF (i.e., via screen use or parental mental health) were significant but nevertheless, the total effect of SES on EF (i.e., including indirect effects via parental mental health and screen use) was significant in both models (Model 1: *β* = 0.191, *p* = 0.017; Model 2: *β* = 0.126, *p* = 0.048). There was no significant direct effect of social support on EF, but social support did show a moderate negative association with parental mental health difficulties and the hypothesized indirect effect of social support on EF via parental mental health was significant for Model 2 (only). The hypothesized indirect effect of social support on EF via enriching activities was not significant, but the total effect of social support on EF (i.e., including indirect effects via parental mental health and enriching activities) was significant for Model 1 (only) (Model 1: *β* = 0.161, *p* = 0.042; Model 2: *β* = 0.106, *p* = 0.091).

**TABLE 3:** Direct and indirect effects of SES and social support on child outcomes, via screen use, parental mental health, and enriching activities after controlling for age and sex

Regression pathways (direct effects)		Model 1: 24‐to‐36‐month‐olds, *n* = 177				Model 2: 24‐to‐51‐month‐olds, *n* = 280			
Independent variable	Dependent variable	*β*	*b*	SE	*p*	*β*	*b*	SE	*p*
SES	Parental mental health	−0.029	−0.029	0.072	0.686	−0.047	−0.047	0.057	0.411
SES	Screen use	**−0.413**	**−0.384**	**0.063**	**<0.001**	**−0.332**	**−0.332**	**0.055**	**<0.001**
SES	EF^a^	0.143	0.085	0.049	0.081	0.088	0.024	0.017	0.168
SES	Externalizing problems	**−0.239**	**−0.066**	**0.024**	**0.007**	**−0.137**	**−0.041**	**0.019**	**0.032**
SES	Internalizing problems	−0.060	−0.016	0.023	0.485	−0.033	−0.010	0.019	0.618
SES	Prosocial behavior	−0.038	−0.013	0.031	0.685	0.002	0.001	0.026	0.978
Social support	Parental mental health	**−0.378**	**−0.397**	**0.079**	**<0.001**	**−0.340**	**−0.340**	**0.059**	**<0.001**
Social support	Enriching activities	0.075	0.074	0.073	0.306	0.088	0.088	0.060	0.141
Social support	EF	0.120	0.076	0.052	0.141	0.045	0.012	0.018	0.482
Social support	Externalizing problems	**−0.183**	**−0.053**	**0.027**	**0.045**	−0.090	−0.027	0.020	0.178
Social support	Internalizing problems	−0.157	−0.045	0.028	0.107	−0.041	−0.012	0.021	0.565
Social support	Prosocial behavior	**0.245**	**0.085**	**0.031**	**0.006**	**0.237**	**0.089**	**0.026**	**0.001**
Parental mental health	EF	−0.081	−0.049	0.047	0.296	**−0.162**	**−0.044**	**0.018**	**0.013**
Parental mental health	Externalizing problems	**0.179**	**0.049**	**0.025**	**0.044**	**0.297**	**0.089**	**0.023**	**<0.001**
Parental mental health	Internalizing problems	0.130	0.035	0.025	0.158	**0.286**	**0.083**	**0.022**	**<0.001**
Enriching activities	EF	**0.131**	**0.084**	**0.039**	**0.032**	0.064	0.018	0.013	0.161
Enriching activities	Prosocial behavior	**0.249**	**0.087**	**0.031**	**0.005**	**0.173**	**0.065**	**0.024**	**0.008**
Screen use	EF	−0.110	−0.070	0.042	0.094	−0.091	−0.025	0.014	0.072
Screen use	Prosocial behavior	−0.087	−0.031	0.032	0.343	−0.029	−0.076	0.026	0.272

Indirect effects										
Independent variable	Mediator	Dependent variable								
SES	Parental mental health	EF	0.002	0.001	0.004	0.706	0.008	0.002	0.003	0.434
SES	Screen use	EF	0.045	0.027	0.017	0.106	0.030	0.008	0.005	0.084
SES	Parental mental health	Externalizing	−0.005	−0.001	0.004	0.692	−0.014	−0.004	0.005	0.421
SES	Parental mental health	Internalizing	−0.004	−0.001	0.003	0.697	−0.013	−0.004	0.005	0.421
SES	Screen use	Prosocial behavior	0.036	0.012	0.013	0.349	0.025	0.010	0.009	0.280
Social support	Parental mental health	EF	0.031	0.019	0.019	0.302	**0.055**	**0.015**	**0.007**	**0.021**
Social support	Enriching activities	EF	0.010	0.006	0.007	0.356	0.006	0.002	0.002	0.310
Social support	Parental mental health	Externalizing	−0.068	−0.020	0.010	0.059	**−0.101**	**−0.030**	**0.009**	**0.001**
Social support	Parental mental health	Internalizing	−0.049	−0.014	0.010	0.170	**−0.097**	**−0.028**	**0.009**	**0.001**
Social support	Enriching activities	Prosocial behavior	0.019	0.006	0.007	0.335	0.015	0.006	0.004	0.197

*Note*: *β* = standardized regression coefficient; *b* = unstandardized regression coefficient. Significant effects (*p* < 0.05) indicated in bold.

Abbreviation: EF, executive functions.

^a^ EF (executive function) latent variable computed using EEFQ CEF items for Model 1, and BRIEF‐P EMI items for Model 2, reverse coded for comparability with Model 1.

Model 1 explained 19% of the variance in child externalizing problems, and Model 2, 17%. There was a small‐to‐moderate positive effect of parental mental health difficulties on externalizing problems in both models, a small negative direct effect of SES on externalizing problems in both models, and a small negative direct effect of social support on externalizing problems in Model 1 (also significant for Model 2 when only 2‐ to 3‐year‐olds were included; see SM 1.5). The hypothesized indirect effect of SES on externalizing problems via parental mental health was not significant in either model, but there was a significant indirect effect of social support on externalizing problems in Model 2 (full sample only). In both models, there was a significant overall total effect of SES (Model 1: *β* = −0.244, *p* = 0.007; Model 2: *β* = −0.151, *p* = 0.023), and social support (Model 1: *β* = −0.251, *p* = 0.007; Model 2: *β* = −0.191, *p* = 0.006) on externalizing problems.

Model 1 explained 9% of the variance in child internalizing problems, and Model 2, 10%. There was a small‐to‐moderate positive effect of parental mental health difficulties on internalizing problems in Model 2 (only). There was no significant direct effect of SES or social support on internalizing problems, and no significant indirect effect of SES on internalizing problems via parental mental health, but there was a significant indirect effect of social support on internalizing problems via parental mental health for Model 2 (which did not reach significance when only 2‐ to 3‐year‐olds were included; see SM 1.5). In both models, there was a significant overall total effect of social support (Model 1: *β* = −0.207, *p* = 0.027; Model 2: *β* = −0.138, *p* = 0.047)—but not SES (Model 1: *β* = −0.064, *p* = 0.460; Model 2: *β* = −0.046, *p* = 0.500)—on internalizing problems.

In the light of the strong‐to‐moderate association between EF and internalizing and externalizing problems, we ran partial correlations to identify whether associations between parental mental health and socio‐emotional difficulties might be attributable to EF. As shown in Table 4, the associations between parental mental health and externalizing and internalizing problems were significant after controlling for EF, or each other.

**TABLE 4:** Partial correlations between aspects of the home environment with child cognitive and socio‐emotional outcomes, controlling for other outcomes

	Controlling for	Externalizing problems		Internalizing problems	
		*r*	*p*	*r*	*p*
Parental mental health	EEFQ CEF (df = 170)	0.229	0.002	0.169	0.027
	BRIEF‐P EMI (df = 277)	0.229	<0.001	0.219	<0.001
	Externalizing/internalizing problems (df = 277)	0.205	0.001	0.240	<0.001

		Prosocial behavior	
		*r*	*p*
Enriching activities	EEFQ CEF (df = 173)	0.193	0.010
	BRIEF‐P EMI (df = 273)	0.151	0.012

Abbreviations: BRIEF‐P, Behavior Rating Inventory of Executive Function – Preschool Version; CEF, cognitive executive function; EEFQ, Early Executive Functions Questionnaire; EMI, Emergent Metacognition Index.

Model 1 explained 20% of the variance in prosocial behavior, and Model 2, 16%. In both models, there was a small‐to‐moderate positive association between enriching activities and prosocial behavior, no significant association between screen use and prosocial behavior, a small‐to‐moderate significant direct effect of social support on prosocial behavior, and no significant direct effect of SES on prosocial behavior. None of the hypothesized indirect pathways from SES and social support to prosocial behavior via screen use or enriching activities were significant. In both models, there was a significant overall total effect of social support (Model 1: *β* = 0.263, *p* = 0.004; Model 2: *β* = 0.252, *p* < 0.001)–but not SES (Model 1: *β* = −0.002, *p* = 0.977; Model 2: *β* = −0.027, *p* = 0.681)–on prosocial behavior.

Given the moderate correlation between EF and prosocial behavior, we ran partial correlations to identify whether associations between enriching activities and prosocial behavior might be attributable to EF. As shown in Table 4, the associations between enriching activities and prosocial behavior were significant after controlling for EF.

### 3.3 Moderation analyses

**FIGURE 4:**
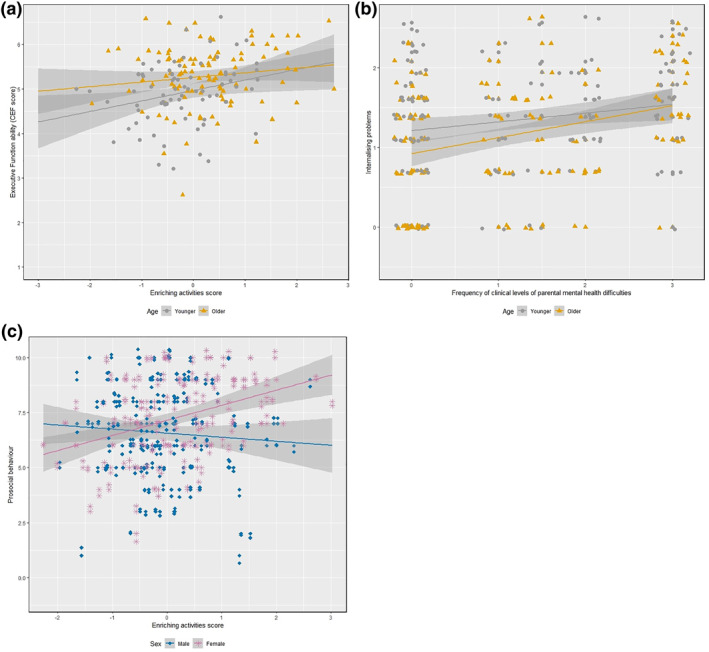
(a) Association between enriching activities and Cognitive Executive Function (CEF) scores, by age (median split). (b) Association between parental mental health and child internalizing problems, by age (median split). (c) Association between enriching activities and child prosocial behavior, by sex

There was a significant interaction effect of enriching activities and child age on CEF; see Table 5. The association between enriching activities and CEF was more pronounced for younger children (cf. Figure 4a). There was also a significant interaction effect of parental mental health and child age on internalizing difficulties. The association between parental mental health and child internalizing difficulties was more pronounced for older children (cf. Figure 4b). Finally, there was a significant interaction effect of enriching activities and child sex on prosocial behavior. The positive association between enriching activities and child prosocial behavior was specific to girls (cf. Figure 4c). No other interaction effects were significant.

**TABLE 5:** Main and interaction effects of aspects of the home environment with child age and sex on child outcomes

Dependent variable	Predictor	*β*	*b*	SE	*p*
CEF^a^	Enriching activities	**0.316**	**0.225**	**0.086**	**0.012**
	Age—Enriching activities	**−0.162**	**−0.114**	**0.055**	**0.033**
	Sex—Enriching activities	−0.054	−0.048	0.108	0.660
	Model *R* ^2^	0.083			
EF difficulties^b^	Parental mental health	**0.213**	**0.044**	**0.016**	**0.008**
	Age—Parental mental health	0.064	0.013	0.012	0.287
	Sex—Parental mental health	−0.038	−0.012	0.025	0.638
	Model *R* ^2^	0.040			
Externalizing difficulties	Parental mental health	**0.333**	**0.185**	**0.043**	**<0.001**
	Age—Parental mental health	0.091	0.048	0.031	0.115
	Sex—Parental mental health	−0.060	−0.050	0.065	0.436
	Model *R* ^2^	0.096			
Internalizing difficulties	Parental mental health	**0.271**	**0.189**	**0.055**	**<0.001**
	Age—Parental mental health	**0.123**	**0.082**	**0.039**	**0.034**
	Sex—Parental mental health	−0.009	0.009	0.082	0.912
	Model *R* ^2^	0.085			
Prosocial behavior	Enriching activities	−0.028	−0.055	0.175	0.754
	Age—Enriching activities	**−0.112**	**−0.216**	**0.114**	**0.059**
	Sex—Enriching activities	**0.276**	**0.737**	**0.232**	**0.002**
	Model *R* ^2^	0.077			

*Note*: *β* = standardized regression coefficient; *b* = unstandardized regression coefficient. Significant effects (*p* < 0.05) are indicated in bold.

Abbreviation: EF, executive functions.

^a^ Using CEF composite score (subsample aged 3 years and under only).

^b^ Using BRIEF‐P‐EMI composite score (full sample).

## 4 DISCUSSION

In this study, we examined how variation in multiple aspects of infants' home environment during the COVID‐19 pandemic has impacted infants' cognitive, social, and emotional functioning, and the broader contextual factors that are linked to variation in the home environment. By using a theory‐driven model to examine multiple dimensions of the home environment and child outcomes simultaneously, we have been able to identify distinct developmental pathways that are not only informative to our general understanding of cognitive development but also helpful to target and shape remediative support for children and families negatively impacted by the pandemic.

Our results indicate that parental (predominantly mothers') mental health, parent‐child activities, and child screen use are associated with different aspects of children's cognitive and psycho‐social development, but also that parental mental health, in particular, exerts influence over perceived outcomes in a range of domains. Below, we detail the key pathways implicated in the emergence of executive function difficulties, externalizing and internalizing problems, and prosocial behavior in our sample, and outline implications for policy and practice.

### 4.1 Support‐threat pathway

The results of our path analysis indicate that, consistent with previous research, one way in which child outcomes have been influenced by the home environment is through parental mental health, which in turn is influenced by parents' perceived social support. Across our sample, we observed small‐to‐moderate positive associations between the number of timepoints at which parents reported clinically elevated levels of anxiety, depression, or stress (collected at 3 6‐monthly intervals between Spring 2020 and 2021), and parent report of child externalizing and internalizing problems, and executive function difficulties, collected in Spring 2021. We speculate, based on the literature discussed in the introduction, that mediating mechanisms for these associations between parental mental health and child psycho‐social outcomes may include parental sensitivity and parental harshness; but we are not able to directly address this question with the current dataset.

Associations between parental mental health and child externalizing and internalizing problems held after controlling for executive function (both when operationalized in terms of strengths and difficulties), and for each other, indicating that the impacts of parental mental health ripple across multiple aspects of psycho‐social development rather than being specific to one primary domain which influences other behaviors.

Our results varied somewhat with age. Post hoc moderation analyses indicated that the association between internalizing difficulties and parental mental health was stronger for older children compared with younger children. This may indicate that older preschoolers are more vulnerable to the impacts of chronic parental mental health difficulties compared with toddlers, or it may be that older children have had more exposure to the negative effects of chronic mental health (i.e., before the start of our observation). However, it may also be the case that our measures of externalizing and internalizing problems are less sensitive to emergent mental health difficulties in young toddlers (Patel et al., 2021). We did not find an association between parental mental health and parent report of child executive function difficulties when only children aged 24–36 months were included. While this may in part be attributable to loss of power, the effect size was also very weak; yet there was no significant interaction effect between parental mental health and child age on child executive function difficulties across the sample. Note too that the observed association between executive function and parental mental health was specific to our measure of executive function difficulties relating to memory, planning, and organization (the BRIEF‐P Emergent Metacognition Index) and was not found when child executive functions were operationalized in terms of strengths in inhibitory control, working memory, and cognitive flexibility (EEFQ Cognitive Executive Function). Planning and organization skills are more complex, later‐developing executive functions (hence they are not included in the EEFQ, which is designed for younger toddlers) (Hendry & Holmboe, 2021). Coupled with the age effects outlined above, this may indicate that parental mental health is particularly implicated in the development of later‐maturing neural mechanisms, which support complex executive function development from around 3 years on. Alternatively, it may be that the impacts of parental mental distress on executive functions are specific to those aspects that are particularly socially scaffolded and influenced by modeled behaviors (i.e., planning and organization) (Hoffman et al., 2006). Another possibility is that there may be a step change in the tendency for parents to observe (and potentially overestimate) the extent of their child's difficulties once their child reaches 3 years. In the UK, this is the age at which all children are entitled to at least 15 h a week of access to Early Childhood Education and Care and may be a transition point at which parents begin to worry more about their child's skills and behavior being at a similar level to their peers.

Likely, some of the relations observed between parental mental health and child outcomes are partly bidirectional, with child negative affect or behavioral difficulties eliciting increase in stress, anxiety, and negative mood in parents (Roubinov et al., 2022; Stein et al., 2014). Further, the observed associations between parental mental health and perceived social support in our study may be somewhat reciprocal—that is, parents who are stressed, anxious, or depressed may give lower ratings of perceived social support compared with a mentally well peer in similar circumstances. Similarly, some of the association observed between our measures of parental mental health and child outcomes may be attributable to a tendency for parents to rate their child's behavior more negatively when distressed (Goodman et al., 2011). Our differential profile of results for executive function indicates that this may be particularly true for parental reports of perceived behavioral problems rather than cognitive strengths. However, since parental perceptions of children's ability and behavior also influence long‐term outcomes (Pomerantz et al., 2005; Räty & Kasanen, 2013), this does not undermine the argument that parental mental health difficulties during the COVID‐19 pandemic are implicated in children's outcomes.

### 4.2 Enrichment‐deprivation

In terms of an enrichment‐deprivation pathway, we found a weak positive association between parent‐child enriching activities and child executive functions, when operationalized in terms of strengths in inhibitory control, working memory, and cognitive flexibility. Previous work using a partially overlapping sample has demonstrated an association between parent‐child enriching activities during the first 6 months of the pandemic (the same variable used in this study) and child executive functions measured in Winter 2020 (Hendry et al., 2022); here, we demonstrate that this predictive association remains when executive function is measured around 6 months later, and is most pronounced for younger toddlers. As we did not measure parent‐child activities in the home at the Spring 2021 data collection point, we are unable to deduce whether the consistent association found here is attributable primarily to the home environment in infancy influencing later development, or to continuity in the degree to which parents engage in enriching activities with their child. In either case, however, our data is consistent with the argument that by playing, talking, and engaging with their children when they are babies or toddlers, parents can boost their child's executive function development—although of course any causal interpretation of this observed association should be treated as preliminary in the absence of experimental manipulation.

We also found that children who spent more time engaged in enriching activities with their parents during the first 6 months of the pandemic were reported by their parents to show greater social competence (higher prosocial behavior scores) 6 months later. This finding is consistent with prior research linking cognitive stimulation to social competence in preschoolers (McGroder, 2000). The enriching activities‐prosocial behavior association remained after controlling for executive function, indicating again that the impacts of parent‐child interactions ripple across multiple aspects of psycho‐social development rather than being specific to one primary domain. Follow‐up moderation analysis indicated that the enriching activities‐prosocial behavior association was specific to girls. This may be attributable to gendered differences in the skills and behaviors that are reinforced through parent‐child activities (Morawska, 2020), or to the influence of gendered perceptions of children's prosocial skills (Bouchard et al., 2015). Although in all our models, we observed a direct association between parents' perceived social support and children's social competence, the hypothesized indirect pathway from social support to prosocial behavior via parent‐child enriching activities was not significant. Further research is, therefore, needed to understand the mechanistic processes by which social support is implicated in the development of prosocial behavior, but we speculate that one possible mechanism is that in families where parents have multiple sources of support, the child is more likely to have multiple trusted adults with whom they can interact and learn social competency skills.

In terms of our second hypothesized proximal mechanism within the enrichment‐deprivation pathway, we did not find evidence of a predictive association between screen use during the first 6 months of the pandemic, and either child executive functions or prosocial behavior measured in Spring 2021. This contrasts with our previously reported findings of short‐term negative associations between screen use and child executive functions (Hendry et al., 2022). Again, as we did not measure screen use at the Spring 2021 data collection point, we are unable to deduce whether our results indicate that negative effects of screen use ‘wash out’ over time, perhaps as other factors exert more influence over cognitive and psycho‐social development, or whether after the initial increase in screen use triggered by the pandemic (Bergmann et al., 2021), parents were able to reduce screen time to less potentially impactful levels. It is also possible that use of screen media at a stressful time has some protective long term benefits for child outcomes that balance out the more immediate negative effects observed; for example, by reducing pressure on parental mental health, providing opportunities for joint parent‐child engagement, or by increasing cognitive stimulation when other options for enriching activities were constrained. Follow‐up research using more granular measures of screen use (i.e., broken down by content type, independent vs. parent‐mediated use, or passive vs. active use) may help unpack the potential negative versus positive effects of screen use. In either case, the lack of a negative predictive association between overall screen use and child outcomes may be reassuring for parents—although these null findings should be contextualized within a broader literature linking levels of exposure to media and television in infancy and toddlerhood to longer‐term cognitive and behavioral outcomes (Cliff et al., 2018; McHarg et al., 2020).

### 4.3 Direct and indirect impacts of SES on child outcomes

In both models, there was a significant total effect of SES on executive functions and externalizing problems, in line with an extensive literature linking SES to these child outcomes (Lawson et al., 2018; Peverill et al., 2021). A recent meta‐analysis found that SES‐internalizing associations exist, but are weaker than for SES‐externalizing associations (Peverill et al., 2021). In our study, there was no significant total effect of SES on internalizing difficulties, but as there was less variance in internalizing difficulties compared with externalizing difficulties this may be because internalizing difficulties are less evident in this young age group.

In terms of potential mechanisms linking SES to outcomes in this sample, our results did not indicate a pathway from SES to child outcomes via parental mental health. Further, while there was a significant association between SES and screen use (as previously reported in Hendry et al., 2022), the indirect effect of SES on executive function difficulties via screen use was not significant. Enriching activities were ruled out as a potential mediator of SES‐child outcome associations as previous reports from this sample found that SES was not associated with enriching activities after the first UK lockdown (Hendry et al., 2022). Therefore, we conclude that associations from SES to EF and externalizing difficulties are attributable to some untested mechanism(s). These might include chronic stressors such as crowding, noise exposure, substandard housing, neighborhood violence, parental separation, and family conflict, which are more common for children from disadvantaged backgrounds (Peverill et al., 2021).

### 4.4 Limitations

As previously mentioned, a key limitation of this study is the reliance on parental report across all variables, which increases the likelihood of shared measurement error and the possibility that there may be systematic differences linked to SES or parental mental health in the ways that parents rate their child's behavior and skills. Future research would benefit from using data from other raters (e.g., researcher‐observers, or teachers, or the results of standardized assessments), either as outcome measures or as potential mediators to better understand the mechanisms implicated in the support‐threat pathway, as previously mentioned. Some of this work is already underway, using metrics of parental sensitivity coded from recordings of parent‐child interactions during the first phase of this study (see Davies et al., 2022). And of course, as in any study, each of our metrics is an imperfect simplification: In particular, the duration metrics of enriching activities and screen potentially mask important sources of variation in the quality of these activities (Hendry et al., 2022); and our indicators of SES mask considerable heterogeneity, and may not have the same meaning across nations or racial‐ethnic groups (DeJoseph et al., 2021).

The second limitation relates to the fact that this was a self‐selecting convenience sample of UK parents, with limited representation of families with extremely low SES. Our study demonstrates that the dimensional approach is suitable for understanding the impact of negative experience inputs in the mild‐moderate range: what is missing is data from the most vulnerable in society. This is particularly important as previous reports have highlighted that the most severely disadvantaged groups have experienced the most psychological stressors during the pandemic (Di Gessa et al., 2021; Patel et al., 2020) whereas in our data we did not find a significant association between SES and parental mental health. Further, as 98% of our sample were mothers, our data cannot speak to the specific impacts of paternal mental health on child outcomes, or whether the impacts of parent‐child activities on child development may vary when fathers specifically are considered; further research is merited to consider such questions.

## 5 CONCLUSIONS AND IMPLICATIONS FOR POLICY AND PRACTICE

In this study, we have identified several domains of cognitive and psycho‐social functioning in which young children may show vulnerability due to their experiences during the pandemic. We have highlighted that children whose parents have experienced chronic mental distress will be at elevated likelihood of displaying externalizing behaviors such as disobedience and restlessness, or internalizing behaviors such as clinginess and nervousness, and may be less able to plan and organize their behavior. And we have shown that children whose parents did not engage extensively with them in enriching activities may be less able than their peers to demonstrate social competence, inhibit their impulses, and flexibly interact with the world.

In redressing these difficulties, Early Years practitioners are well‐placed to nurture children in building the skills they will need to thrive (Davies et al., 2021). Settings will need to work with adequately trained practitioners and appropriate ratios (both enabled by sustainable government funding) to provide differentiated support to respond to the needs of individual children across ages, genders, and socioeconomic backgrounds. In addition, a range of universal and targeted intervention programs show good potential for mitigating some of the harms to young children associated with the pandemic (Fox et al., 2021).

To mitigate the likelihood of these negative effects becoming entrenched for this ‘pandemic generation’ of toddlers, and as a preventative measure for those just being born, our data highlight the importance of supporting the mental health of parents of very young children. Given the observed associations between parental mental health and perceived social support, an obvious focus for intervention is to support parents in building and accessing support networks. This is likely to require greater investment in universal services such as health visitors who can provide informational support and signposting to specialist services where necessary, as well as funding for community services, which can provide peer‐to‐peer support to parents who are struggling. If these networks are also able to support parents in engaging in enriching activities with their child, then our research suggests that the benefits for children are likely to be spread across many aspects of cognitive and psycho‐social development.

## CONFLICT OF INTEREST

The authors declare no conflicts of interest with regard to the funding source for this study.

## Supporting information

Supporting Information S1
