# Supplementary material for: Toward a dimensional model of risk and protective factors influencing children's early cognitive, social, and emotional development during the COVID‐19 pandemic
Source: Infancy. 2022 Aug 22;28(1):158–86. doi: 10.1111/infa.12495 (PMC10086814; doi:10.1111/infa.12495)
Supplement: Supplementary file 1 — Supporting Information S1 [file INFA-28-158-s001.docx]

Supplementary Materials 1: Further sample and variable details and follow-up analyses

# 1. 1 Characteristics of participants at each wave of the study

*Supplementary Table 1.11.* Profile of participants at each wave of data collection, with regard to SES characteristics. Cells show mean scores with standard deviation in round parentheses and minimum and maximum scores in square parentheses.

| Sample | n | Neighbourhood deprivation^1^ | Household Income^2^ | Parental Education^3^ | Parental Occupation^4^ |
| --- | --- | --- | --- | --- | --- |
| Original valid sample* | 861 | 6.78 (2.35)  [1,10] | 4.90 (1.91)  [1,7] | 5.24 (1.24)  [2,8] | 6.85 (1.62)  [2,9] |
| With Spring 2020 data | 502 | 6.92 (2.29)  [1,10] | 4.96 (1.91)  [1,7] | 5.38 (1.25)  [2,8] | 7.01 (1.54)  [3,9] |
| With Winter 2020 data | 227 | 6.76 (2.60)  [1,10] | 4.96 (1.88)  [1,7] | 5.45 (1.26)  [2.5,8] | 7.07 (1.56)  [3,9] |
| With Spring 2021 data | 280 | 6.71 (2.64)  [1,10] | 4.85 (1.91)  [1,7] | 5.38 (1.29)  [2,8] | 6.82 (1.56)  [3,9] |

^*^Provided demographic data in Spring 2020 but did not necessarily contribute the rest of the Spring 2020 questionnaires.

^1^ Index of Multiple Deprivation decile, where 1= most deprived, 10 = least deprived
^2^ Household income brackets: 1=£0-£20k, 2=£21k-£30k, 3=£31k-£40k, 4=£41k-£50k, 5=£51k-£60k, 6=£61k-£70k, 7=£71k or over
^3^ Categories of highest level of education completed: 1= Primary school, 2=Secondary school, 3=Sixth form or college, 4=Vocational college, 5=Undergraduate, 6=Postgraduate, 7=MBA, 8= Doctoral degree
^4^ Occupational prestige where 1 = lowest prestige, 9 = highest prestige

Consistent with previous work on SES and cognitive development, we conceptualise SES as a formative latent variable (Figlio, Freese, Karbownik, & Roth, 2017; Ramphal et al., 2020; Smith, Kievit, & Astle, 2021). Therefore, to minimise the number of comparisons required, Principal Components Analysis (PCA) was reduce the data from our 4 indices of SES (neighbourhood deprivation, household income, parental education, and parental occupation) (Filmer & Pritchett, 2001; Vyas & Kumaranayake, 2006). Only one PCA factor had an eigenvalue over Kaiser’s criterion of 1. This factor, which we labelled SES, explained 55% of the variance. SES factor loadings are shown in Supplementary Table 1.2 and indicate that levels of cultural capital (education and occupational status) and economic capital (income and deprivation) are intertwined in our sample. The extracted SES factor scores were used in all the analyses reported in the main manuscript.

*Supplementary Table 1.12.* PCA factor loadings

|  | SES factor loadings |
| --- | --- |
| Parental Occupation Score | .828 |
| Household Income | .823 |
| Parental Education Score | .781 |
| Neighbourhood deprivation index | .528 |

# 1. 2 Enriching activities and screen use

In Spring 2020, respondents were asked to report on the kinds of activities that they did with their child – for example reading, singing, arts and crafts, cooking and baking; see Supplementary Table 1.2 for details. Parents were asked to estimate how much time on average, they spent together with their child doing the activities listed in the first column of Supplementary Table 1.2. Parents were also asked to report on the amount of time that their child did some of these activities alone, but only parent-child activities are included in this study. Items were categorised as indicated in the second column of Supplementary Table 1.2 (these headings were not displayed to respondents).

Questions were based on a home activities measure developed to investigate the effects of Covid-19 lockdowns on language development in different countries (Kartushina et al., 2021), and a screen-use measure developed to investigate changes in and impacts of infant screen use in different countries (Bergmann et al., 2021). For the Spring 2020 questionnaire, the respondents reported on a scale of 0 (“Did not do at all”) to 9 (“Performed this activity more than 4 hours most days”. We calculated an Enriching Activities score by summing the score for each enriching activity item carried out with a parent during the Spring 2020 lockdown period (parents were also asked to report on activity prior to lockdown; this data is not used here). Cronbach’s alpha for the 11-item Spring 2020 Enriching Activities scale =.706. We calculated a Screen Use score by summing the score for each of the 6 activity items that involved watching TV or playing on a touchscreen. Cronbach’s alpha for the Spring 2020 Screen Use scale =.648.

In Winter 2020, respondents were again asked to report on the kinds of activities that their child spent time doing, but for increased granularity respondents were asked first to report how many days per week they did each activity on a scale of 0-7, and then to estimate how much time per day on average was spent on each activity on a scale of 1 (0-15 minutes) to 7 (more than 4 hours). These values were multiplied to compute a total for each activity on a scale of 0-49 and then summed to compute a Winter 2020 Enriching Activities Score. We calculated a Winter lockdown Screen Use score by summing the score for each of the 6 activity items that involved watching TV or playing on a touchscreen. Cronbach’s alpha for the 12-item Winter lockdown Enriching Activities scale =.815. Cronbach’s alpha for the Winter lockdown Screen Use score scale =.709. Winter 2020 Enriching Activities scores were moderately correlated with Spring 2020 Enriching Activities scores (*r*=.415, *p*<.001). A 2020 Pandemic Enriching Activities score (henceforth ‘Enriching activities score’) was computed by standardising Spring 2020 and Winter 2020 Enriching Activities scores and computing the mean. Winter 2020 Screen Use scores were highly correlated with Spring 2020 Screen Use scores (*r*=.616, *p*<.001). A 2020 Pandemic Screen Use score (henceforth ‘Screen us score’) was computed by standardising Spring lockdown and Winter lockdown Screen Use scores and computing the mean.

*Supplementary Table 1.2* Enriching activities items and screen use items

| Item | Usage |
| --- | --- |
| Reading a (child) book with your child (or to your child) | Enriching Activities scale |
| Playing organised games with a specific learning goal (e.g. flashcards, counting, board-games, puzzles, etc.) | Enriching Activities scale |
| Free-play with your child (e.g. building blocks/railways, playing with dolls, cars racing, lego, etc.). | Enriching Activities scale |
| Singing with your child (e.g. children's songs) | Enriching Activities scale |
| Direct one-to-one speaking to your child (or parent-child conversations) | Enriching Activities scale |
| Indoor exercise with your child (e.g. dancing, yoga) | Enriching Activities scale |
| Arts and crafts (e.g. colouring, drawing, creating something) with your child | Enriching Activities scale |
| Cooking and baking with your child | Enriching Activities scale |
| Gardening (e.g. digging, planting seeds) with your child | Enriching Activities scale |
| Outdoor exercise (e.g. football, walks, biking) with your child | Enriching Activities scale |
| Non-active shared time outside (e.g. picnic, watching the clouds) with your child | Enriching Activities scale |
| Eating meals together without the TV on with your child | Enriching Activities scale  (subsequently dropped)^a^ |
| Helping with household tasks (e.g. cleaning windows, tidies up the house) | Enriching Activities scale: Winter 2020 only ^b^ |
| Watching baby cartoons/shows/TV with your child | Screen Use scale |
| Watching cartoons/shows/TV made for other viewers older than your child (e.g. older siblings or adults) with your child | Screen Use scale |
| Virtual interactions with other adults (e.g. Skype) with your child | Not used (excluded from AAP screen guidelines) |
| Virtual interactions with other children (e.g. Skype) with your child | Not used (excluded from AAP screen guidelines) |
| Playing baby games on a digitalised support (for example, on Smartphone/Tablet) with your child | Screen Use scale |
| Free-play without an adult (e.g. your child plays on their own or with siblings, for example, lego, blocks, puzzles, dolls, cars, etc.) | Not used (to retain focus on adult-led enriching activities) |
| Indoor exercise (e.g. dancing, yoga without an adult | Not used (as above) |
| Arts and crafts (e.g. colouring, drawing, creating something) without an adult | Not used (as above) |
| Outdoor exercise (e.g. football, walks, biking) without an adult | Not used (as above) |
| Gardening (e.g. digging, planting seeds) without an adult | Not used (as above) |
| Non-active time outside (e.g. picnic, watching the clouds) without an adult | Not used (as above) |
| Watching baby cartoons/shows/TV without an adult | Screen Use scale |
| Watching cartoons/shows/TV made for other viewers older than your child (e.g. older siblings or adults) without an adult | Screen Use scale |
| Playing baby games on a digitalised support (for example, on Smartphone/Tablet) without an adult | Screen Use scale |

^a^ Not included in the Enriching activities score due to low internal consistency with the other items.
^b^ Added to the Winter 2020 questionnaire, as this had been frequently mentioned by parents as an ‘Other’ activity at the previous timepoint.

# 1. 3 SDQ scores contextualised with regards to previously-published data

This was a self-selective sample, with a skew towards high SES, and therefore comparisons with previously collected data should be treated with caution and cannot be used to draw strong conclusions about the impact of the pandemic in general on children’s psycho-social development. Moreover, comprehensive population-level SDQ age-normed data is not currently available for UK based 2- to 4-year-olds. Nevertheless, documenting how scores compare with previously-collected data from broadly-equivalent populations may provide a useful context for interpreting the data presented in the main manuscript. Thus, Supplementary Table 1.1 uses provisional banding cut-offs derived from a sample of over 20,000 2- to 4-year-olds in Scotland (Youth in Mind, 2022) whereby cut-off points were selected so that around 80% of children are ‘close to average’, 12% ‘slightly raised’, 4% ‘high’ and 4% ‘very high’ (or, for the prosocial scale, 80% are close to average, 12% ‘slightly lowered’, 4% ‘low’ and 4% ‘very low’).

Supplementary Table 1.3: Proportions of sample showing raised, high and very high SDQ scores, by scale

|  | Close to average | Slightly raised (lowered for prosocial) | High  (low for prosocial) | Very high  (very low for prosocial) |
| --- | --- | --- | --- | --- |
| Comparison sample proportions | 80% | 12% | 4% | 4% |
| SDQ total difficulties | 78.6% | 13.2% | 3.2% | 5.0% |
| Emotional problems ^a^ | 80.0% | 11.8% | 3.2% | 5.0% |
| Peer  problems ^a^ | 71.4% | 9.3% | 6.4% | 12.9% |
| Conduct  problems ^b^ | 80.4% | 11.1% | 4.3% | 4.3% |
| Hyperactivity/ inattention ^b^ | 83.9% | 6.1% | 4.3% | 5.7% |
| Prosocial | 57.1% | 19.3% | 13.2% | 10.4% |

^a^ Part of the Internalising problems subscale
^b^ Part of the Externalising problems subscale

As shown in Supplementary Table 1.1, the proportion of children showing close to average, slightly raised, high, and very high difficulties was broadly comparable to provisional norms developed for the SDQ in terms of total difficulties, emotional problems, conduct problems and hyperactivity. However, there was a marked difference to the provisional SDQ norms in terms of prosocial behaviour, with elevated proportions showing slightly lowered, low and very low prosocial behaviour. There was also a difference to the provisional SDQ norms in terms of peer problems, with 3 times as many as expected showing very high problems.

# 1. 2 BRIEF-P scores contextualised with regards to previously-published data

As above, because this was a self-selective sample with a skew towards high SES, comparisons with previously collected data cannot be used to draw strong conclusions about the impact of the pandemic in general on children’s cognitive development. Moreover, published norms for the BRIEF-P are based on a US sample, screened for developmental difficulties, attention problems and other cognitive difficulties (Gioia, Espy, & Isquith, 2002). Nevertheless, to aid with interpreting the data, raw EMI scores (which were used for all key analyses) were translated into T scores using the published conversion algorithms appropriate to each participant’s sex and age, whereby 50 represents the mean of the standardardized T score distribution, and a T score of 65 represents 1.5 standard deviations above the mean, which is the recommended threshold for interpretation of a score as showing clinically significant difficulties. According to these criteria, 16.4% of participants scored in the clinically significant range for EF difficulties on the Emergent Metacognition index.

# 1. 4 Structural regression

The structural model was specified with separate latent factors for EF (with loadings from EEFQ CEF for Model 1 and BRIEF-P EMI items for Model 2), externalising difficulties (SDQ conduct problems and hyperactivity/inattention items), internalising difficulties (SDQ emotional symptoms and peer relationship problems items), and prosocial behaviour (SDQ prosocial behaviour items). These models showed good fit (Model 1 RMSEA = 0.056, SRMR = 0.066; Model 2 RMSEA = 0.059, SRMR = 0.067), and were better than a model in which all items were loaded onto a single factor (Model 1 comparison Χ^2^(6)=596.4, *p*<.001; Model 2 comparison Χ^2^(6)=547.09, *p*<.001). However, in Model 1 EEFQ items WM6 and WM game showed low loadings onto the CEF factor (.055 and .214 respectively), and were therefore removed from the structural model.

# 1.5 Path analysis using Model 2 but including only 2- to 3-year-olds

*Supplementary Table 1.5.* Direct and indirect effects of SES and social support on child outcomes, via screen use, parental mental health and enriching activities after controlling for age and sex

| Regression pathways (direct effects) | | | | Model 2b 24-to-36-month-olds, *n*=177 | | | | | | | | | |  | |  |
| --- | --- | --- | --- | --- | --- | --- | --- | --- | --- | --- | --- | --- | --- | --- | --- | --- |
| Independent Variable | | **Dependent Variable** | | *β* | | *b* | | SE | |  | | *p* | |  | |  |
| SES |  | | Parental mental health | | -0.029 | | -0.029 | | 0.072 | |  | | 0.688 | |  | |
| SES |  | | Screen use | | **-0.413** | | **-0.384** | | **0.063** | |  | | **<.001** | |  | |
| SES |  | | EF^a^ | | -0.029 | | -0.029 | | 0.072 | |  | | 0.688 | |  | |
| SES |  | | Externalizing problems | | **-0.240** | | **-0.066** | | **0.024** | |  | | **0.007** | |  | |
| SES |  | | Internalizing problems | | -0.060 | | -0.016 | | 0.023 | |  | | 0.483 | |  | |
| SES |  | | Prosocial behaviour | | -0.039 | | -0.013 | | 0.031 | |  | | 0.680 | |  | |
| Social support |  | | Parental mental health | | -0.377 | | -0.396 | | 0.079 | |  | | **<0.001** | |  | |
| Social support |  | | Enriching activities | | 0.075 | | 0.074 | | 0.073 | |  | | 0.306 | |  | |
| Social support |  | | EF | | 0.069 | | 0.020 | | 0.024 | |  | | 0.403 | |  | |
| Social support |  | | Externalizing problems | | **-0.183** | | **-0.054** | | **0.027** | |  | | **0.045** | |  | |
| Social support |  | | Internalizing problems | | -0.156 | | -0.044 | | 0.028 | |  | | 0.108 | |  | |
| Parental mental health |  | | EF | | -0.107 | | -0.029 | | 0.022 | |  | | 0.197 | |  | |
| Parental mental health |  | | Externalizing problems | | **0.180** | | **0.050** | | **0.025** | |  | | **0.043** | |  | |
| Parental mental health |  | | Internalizing problems | | 0.131 | | 0.035 | | 0.025 | |  | | 0.156 | |  | |
| Enriching activities |  | | EF | | 0.074 | | 0.021 | | 0.018 | |  | | 0.228 | |  | |
| Enriching activities |  | | Prosocial behaviour | | **0.249** | | **0.088** | | **0.031** | |  | | **0.005** | |  | |
| Screen use |  | | EF | | -0.092 | | -0.027 | | 0.019 | |  | | 0.171 | |  | |
| Screen use |  | | Prosocial behaviour | | -0.087 | | -0.031 | | 0.032 | |  | | 0.342 | |  | |
| *Indirect effects* |  | |  | |  | |  | |  | |  | |  | |  | |
| Independent Variable | **Mediator** | | **Dependent Variable** | |  | |  | |  | |  | |  | |  | |
| SES | Parental mental health | | EF | | 0.003 | | 0.001 | | 0.002 | |  | | 0.701 | |  | |
| SES | Screen use | | EF | | 0.038 | | 0.010 | | 0.008 | |  | | 0.182 | |  | |
| SES | Parental mental health | | Externalizing | | -0.005 | | -0.001 | | 0.004 | |  | | 0.694 | |  | |
| SES | Parental mental health | | Internalizing | | -0.004 | | -0.001 | | 0.003 | |  | | 0.699 | |  | |
| SES | Screen use | | Prosocial behaviour | | 0.036 | | 0.012 | | 0.013 | |  | | 0.348 | |  | |
| Social support | Parental mental health | | EF | | 0.040 | | 0.011 | | 0.009 | |  | | 0.205 | |  | |
| Social support | Enriching activities | | EF | | 0.006 | | 0.002 | | 0.002 | |  | | 0.435 | |  | |
| Social support | Parental mental health | | Externalizing | | -0.068 | | -0.020 | | 0.010 | |  | | 0.057 | |  | |
| Social support | Parental mental health | | Internalizing | | -0.049 | | -0.014 | | 0.010 | |  | | 0.169 | |  | |
| Social support | Enriching activities | | Prosocial behaviour | | **0.245** | | **0.085** | | **0.031** | |  | | **0.006** | |  | |

*β* = standardized regression coefficient b = unstandardized regression coefficient. Significant effects (*p*<.05) indicated in bold.
^a^ EF (executive function) latent variable computed using EEFQ CEF items for Model 1, and BRIEF-P EMI items for Model 2, reverse coded for comparability with Model 1.

**References**

Bergmann, C., Dimitrova, N., Alaslani, K., Almohammadi, A., Alroqi, H., Aussems, S., . . . Mani, N. (2021). Young children's screen time during the first COVID-19 lockdown in 12 countries. *OSF*. <https://doi.org/10.31219/osf.io/p5gm4>

Figlio, D. N., Freese, J., Karbownik, K., & Roth, J. (2017). Socioeconomic status and genetic influences on cognitive development. *Proceedings of the National Academy of Sciences, 114*(51), 13441-13446. doi:10.1073/pnas.1708491114

Filmer, D., & Pritchett, L. H. (2001). Estimating wealth effects without expenditure data—or tears: an application to educational enrollments in states of India. *Demography, 38*(1), 115-132.

Gioia, G. A., Espy, K. A., & Isquith, P. K. (2002). *Behavior Rating Inventory of Executive Function - Preschool Version*. Odessa, FL: Psychological Assessment Resources.

Ramphal, B., Whalen, D. J., Kenley, J. K., Yu, Q., Smyser, C. D., Rogers, C. E., & Sylvester, C. M. (2020). Brain connectivity and socioeconomic status at birth and externalizing symptoms at age 2 years. *Developmental Cognitive Neuroscience, 45*, 100811. <https://doi.org/10.1016/j.dcn.2020.100811>

Smith, T. A., Kievit, R., & Astle, D. (2021). Parental Mental Health Mediates Links Between Socioeconomic Status and Child Development. PsyArXiv https://doi.org/10.31234/osf.io/4swy8

Vyas, S., & Kumaranayake, L. (2006). Constructing socio-economic status indices: how to use principal components analysis. *Health Policy and Planning, 21*(6), 459-468. doi:10.1093/heapol/czl029#

Youth in Mind, (28/05/2014). SDQ for 2-4 year olds: Normative data from Britain. https://sdqinfo.org/norms/UK3yearNorm.html
